# In Vitro Response of *Polyscias filicifolia* (Araliaceae) Shoots to Elicitation with Alarmone–Diadenosine Triphosphate, Methyl Jasmonate, and Salicylic Acid

**DOI:** 10.3390/cells10020419

**Published:** 2021-02-17

**Authors:** Anita Śliwińska, Marcin R. Naliwajski, Agnieszka Pietrosiuk, Katarzyna Sykłowska-Baranek

**Affiliations:** 1 Department of Pharmaceutical Biology and Medicinal Plant Biotechnology, Faculty of Pharmacy, Medical University of Warsaw, 1 Banacha, 02-097 Warsaw, Poland; anita.sliwinska@wum.edu.pl (A.Ś.); agnieszka.pietrosiuk@wum.edu.pl (A.P.); katarzyna.syklowska-baranek@wum.edu.pl (K.S.-B.); 2 Department of Plant Physiology and Biochemistry, Faculty of Biology and Environmental Protection, University of Lodz, ul. Banacha 12/16, 90-237 Lodz, Poland

**Keywords:** *Polyscias filicifolia*, elicitation, diadenosine 5′,5‴-*P*^1^*P*^3^-triphosphate, oleanolic acid, phenolic compounds, antioxidant activity, antioxidant enzymes, tocopherol

## Abstract

The effectiveness of different elicitation variants in combination with alarmone application was studied in shoot cultures of *Polyscias filicifolia*. The shoots were elicited with 200 µM methyl jasmonate (MeJA) or 50 µM salicylic acid (SA) alone or in combination, and their activity was compared with those treated with the alarmone diadenosine 5′,5‴-*P*^1^*P*^3^-triphosphate (Ap3A), either alone or in combination with SA and/or MeJA. All treatments resulted in significant stimulation of phenolic acid production (chlorogenic and ferulic acids), as well as oleanolic acid (OA) compared to control, with their highest concentration noted under simultaneous elicitation with SA and MeJA. While the maximum content of caffeic acid was detected after treatment with alarmone alone. In each of the culture variants enhanced antioxidant activity was observed, however the level varied according to the treatment. In addition, the SA, Ap3A and Ap3A+SA variants demonstrated additional peroxidase isoforms, as indicated by Native-PAGE, as well as the highest *α-*tocopherol content. The highest antioxidant capacity of shoot extracts was correlated with the highest abundance of phenolic compounds and OA. The results indicate that ROS induction appears to participate in the signal transduction following Ap3A treatment.

## 1. Introduction

Plant secondary metabolites are a wide and diversified group of low molecular weight compounds. They are known to act as biotic and abiotic stressors and hence play a crucial role in the responses of plants to changing environmental conditions. The diversity of these environmental stress factors has resulted in the development of complex signaling systems which enable efficient defensive strategies by the plant. Plant defence network involves two major signaling pathways: salicylic acid (SA) and jasmonic acid (JA)-dependent that influence each other [1,2,3]. Although the negative cross-talk between SA and JA pathways was reported [4,5], this relation could be shifted from antagonistic to synergistic depending on the concentrations of SA and JA. Such flexibility would enhance the potential for the plant to adapt to a wider range of environmental stimuli [2,5]. Treatment with elicitors has been found to result in the enhanced generation of reactive oxygen species (ROS); these are believed to act as signaling molecules that modulate specific protein activities, gene expression and metabolic fluxes [6,7,8,9]. Nevertheless, the most instant reaction to environmental stress is oxidative burst, which is a huge increase in ROS generation that could exert detrimental effect on cells [10]. The plant defence system, which is launched in response to excessive toxic ROS levels, consists of enzymatic antioxidants such as catalase (CAT), superoxide dismutase (SOD), peroxidase (POX), and glutathione S-transferase (GST) as well as non-enzymatic antioxidants, flavonoids, phenolic acids and anthocyanins [11,12]. In plant preparation to survive under stress conditions also alarmones were reported to be involved [13]. The most common alarmones, Ap3A (diadenosine 5′, 5‴-*P*^1^*P*^3^-triphosphate) and Ap4A (diadenosine 5′,5‴-*P*^1^*P*^4^-tetraphosphate), have been found to stimulate the expression of genes involved in phenylpropanoid pathway when applied exogenously to seedlings of *Arabidopsis thaliana* [13] or to cell suspension cultures of *Vitis vinifera* [14].

The combined application of elicitors known to stimulate different biosynthetic pathways has been found to have a pronounced impact on the productivity of plant in vitro cultures, resulting in significant increase in accumulation of desired metabolites [15,16].

*Polyscias filicifolia* (C. Moore ex E. Fourn.) L. H. Bailey (Araliaceae), an evergreen shrub known as fern leaf Panax, is used in traditional Southeast Asia medicine due to its adaptogenic properties [17], and for its anti-fatigue, immunomodulating and anti-dizziness activity [18]. However, its phytochemical constituents are not fully recognized. It was reported that the leaves and roots are abundant sources of triterpenoid saponins [19], whose presence was also confirmed in other *Polyscias* species [20]. Interestingly, oleanolic acid glycosides have been determined in cell suspension cultures of *P. filicifolia*, but only those established from callus developed directly on leafy explants. Oleanolic acid (OA), an aglycone of triterpene glycosides, is a pentacyclic triterpenoid compound that is widespread in the plant kingdom, including *Polyscias* genus [21]. OA was demonstrated to possess a wide range of hepatoprotective, anti-inflammatory, antioxidant and anticancer and cardioprotective properties [21,22,23], as well as antibacterial and antiparasitic effects [24]. Due to the significant pharmacological potential of OA, various biotechnological approaches have been designed to enhance its yield in in vitro cultures but none of them concerns shoot cultures [25]. Although *P. filicifolia* can be harvested from natural sources, or even cultivated in tropical areas, in vitro cultures allow more efficient production by avoiding the slow growth of the plants and the need for specific environmental conditions. In addition, the use of such biotechnological approaches can maintain biological diversity.

Only a limited range of biotechnological approaches have been developed for in vitro secondary metabolites production in *P. filicifolia* cultures. It has been reported recently that shoots of *P. filicifolia* accumulated significant amounts of phenolic compounds, in response to elicitation with methyl jasmonate (MeJA) or salicylic acid (SA), with chlorogenic acid predominating [26]. Furthermore, the application of combined elicitation with 200 µM MeJA and 50 µM SA improved phenolic compounds yield [27]. Interestingly, the extracts derived from the elicited shoots demonstrated more potent antigenotoxic, anti-photogenotoxic and antioxidant activity than the standard compounds, *viz*. chlorogenic (CGA), caffeic (CA) and ferulic (FA) acids. Furthermore, the chemical analysis also revealed the presence of OA among other constituents in investigated extracts. These very promising results, served as the foundation for further biotechnological studies which aimed to enhance the biosynthesis of biologically active compounds in shoot cultures of *Polyscias filicifolia*.

The present study examines a new approach based on the combined application of the Ap3A alarmone together with two elicitors MeJA and SA to enhance the production of phenolic compounds and OA in *Polyscias filicifolia* shoot cultures. It also examines the possible role of ROS in signal transduction in producing the desired compounds following elicitation with Ap3A, and determines the antioxidant potential of the resulting plant extracts.

## 2. Materials and Methods

### 2.1. Chemicals and Reagents

All chemicals and reagents, unless stated otherwise, were purchased from Sigma-Aldrich (Poznań, Poland) or in Avantor Performance Materials Poland S.A. (Gliwice, Poland). Standard compounds of oleanolic acid, caffeic acid, chlorogenic acid and ferulic acid, as well as diadenosine 5′,5‴-*P^1^P^3^*-triphosphate were purchased from Sigma-Aldrich (Poznań, Poland).

### 2.2. Shoot Cultures

The studied explants were *P. filicifolia* plantlets developed as described by Śliwińska et al. [28]. The plantlets were cultured on 1/3 strength MS [29] solid medium without growth regulators for three months. Following this, their apical parts were isolated (3.5–4.5 cm length) and multiplied in the first stage on LS [30] solid medium supplemented with 8.88 µM (2 mg/L) 6-benzylaminopurine (BAP) and 2.32 µM (0.5 mg/L) kinetin (KIN), 30 g/L sucrose and cultivated for five weeks at 16 h/8 h (light/dark) photoperiod with light provided by cool-white fluorescent lamps (40 µM/m^2^/s^1^) at 25 ± 1 °C [28]. BAP and KIN were added to the medium prior to autoclaving. Next, the experiments with medium supplementation with methyl jasmonate (MeJA), salicylic acid (SA) and diadenosine 5′,5″-*P*^1^*P*^3^-triphosphate (Ap3A) were performed. The concentration of the two elicitors, were chosen on the basis of Figat et al. [27], as the most effective for stimulation secondary metabolite biosynthesis in *P. filicifolia* shoot cultures. Ap3A concertation was chosen based on Pietrowska-Borek et al. [14]. MeJA, SA and Ap3A were added to the medium after autoclaving. The five-week old shoots were placed on the following modifications of LS medium: (i) LS without elicitors – control (LS0); (ii) LS + 50 µM SA; (iii) LS + 200 µM MeJA; (iv) LS + 50 µM SA and 200 µM MeJA; (v) LS + 5 µM Ap3A; (vi) LS + 5 µM Ap3A and 50 µM SA; (vii) LS + 5 µM Ap3A and 200 µM MeJA; (viii) LS + 5 µM Ap3A and 50 µM SA + 200 µM MeJA. They were then cultured for one week.

All shoot cultures were performed in 300 mL Erlenmeyer flask containing 60 mL of solid medium. All media were adjusted to pH 5.7 before adding 8 g/L plant Propagation Agar (Biocorp, Warsaw, Poland) and sterilized by autoclaving at 121 °C for 20 min. All experiments were conducted in three replications, each consisting of four flasks with four explants. N = 12 for control and each treatment. After one week, the explants were collected, the number of shoots and their length were recorded. Next their fresh weight (FW) and dry weight (DW) after lyophilization were determined. The increase in shoot biomass was calculated as a ratio of final weight to the initial weight of shoots. Shoot multiplication rate was calculated as a ratio of initial shoot number to final shoot number.

### 2.3. Phytochemical Analysis Using HPLC-UV-DAD

#### 2.3.1. Determination of Phenolic Acids

The contents of CA, CGA and FA were determined in methanolic extracts prepared from the shoots of *P. filicifolia* harvested from control cultures and from the seven different alarmone and/or elicitor treatments. Sample preparation and HPLC analysis were performed according to Śliwińska et al. [26]. The lyophilized and powdered plant material (0.1 ± 0.002 g) was extracted with 100% methanol (4 × 10 mL) for 15 min using an ultrasonic bath (Sonorex, Bandelin, Berlin, Germany). Following this, the samples were collected and evaporated to dryness under reduced pressure and stored at −20 °C before analysis. Prior to HPLC-DAD-UV-Vis analysis, the dry residue was dissolved in 100% methanol and subjected to a DIONEX HPLC system (Sunnyvale, California, USA), equipped with an automated sample injector (ASI-100) and UVD 340S detector. The following gradient program was applied: 0 min, B10%; 5 min, B45%; 15 min, B55% was applied and the flow rate was set at 1 mL/min. C18 reversed phase column (EC 250/4.6 Nucleosil 120–127 mm; Macherey-Nagel, Düren, Germany) was used; the data were recorded at 327 nm. Peaks were assigned by spiking the samples with the standards and comparing the retention times and UV spectra.

#### 2.3.2. Determination of Oleanolic Acid

The content of oleanolic acid (OA) was determined in the *P. filicifolia* shoots cultivated under the same conditions of the current experiments. The powdered lyophilized shoots (0.5 ± 0.002 g) were extracted using an ultrasonic bath with 100% methanol (4 × 10 mL) for 30 min. The methanolic extracts containing a mixture of oleanolic acid glycosides were evaporated to dryness. The dry residue was dissolved in 2 mL of methanol and then 10 mL of 1 M HCL was added and the hydrolysis continued for five hours at 80 °C. The hydrolysates were evaporated to dryness, dissolved in 20 mL distilled water and the OA was extracted four times with 10 mL of diethyl ether. The ether extracts were collected and then evaporated to dryness. The dry residue was dissolved in 100% methanol and subjected to HPLC-DAD-UV-Vis analysis on a DIONEX HPLC system as described in Section 2.3.1. The HPLC analysis was performed according to Liang et al. [31]. The extracts were analyzed using an isocratic program: solvent A, 0.2% NH_4_OAc; solvent B, methanol. The mobile phase was a mixture of methanol: water (83:17 containing 0.2% NH_4_OAc), at a flow-rate of 1.5 mL/min, using a C18 reversed-phase column (EC 250/4.6 Nucleosil 120-127 mm; Macherey-Nagel, Düren, Germany). The data were recorded at 210 nm. Peaks were identified by spiking the samples with the standard substance and comparing the retention times and UV spectra.

### 2.4. Determination of Total Flavonoid Content (TFC)

The total flavonoid content in the shoot extracts was determined by as described previously [32] with minor modifications. Appropriately diluted extracts from the shoots of *P. filicifolia* (25 µL) were mixed with 50 µL of 5 % sodium acetate for 5 min. Following this, 100 µL 2% AlCl_3_ was added and mixed, and then 100 µL 1 M NaOH was added. The 96-well plates were incubated in the darkness at room temperature for 15 min. The absorbance of the samples was measured at 425 nm. The results were calculated as the mean value of four replicates for each sample, and expressed as mg of quercetin equivalent (QE) [mg QE/g DW] using the regression equation determined from the standard curve: y = 0.0023x + 0.2383, r^2^ = 0.9921. The Epoch BioTek (Winooski, Vermont, USA) microplate reader was used.

### 2.5. Determination of Total Phenolic Compound Content (TPC)

The total phenolic compound content in the extracts was determined in 96-well plates according to the Folin–Ciocalteu method as described previously [32] with some modifications. Appropriately diluted extracts from the shoots of *P. filicifolia* (25 µL) were mixed with 40 µL of Folin-Ciocalteu reagent. After five minutes, 160 µL sodium carbonate was added and the mixture was incubated in the dark at room temperature for 30 min. The absorbance of the samples was measured at 765 nm. The results were calculated as the mean value of four replicates for each sample, and expressed as mg of gallic acid equivalent (GAE) [mg GAE/g DW] using the regression equation determined from the standard curve: y = 0.0056x + 0.1881, r^2^ = 0.9961.

### 2.6. Enzyme Assays

#### 2.6.1. Determination of Enzymes Activity as well as TBARS and Tocopherol Content

Enzymes activities, Thiobarbituric Acid Reactive Substance (TBARS), protein level and α-tocopherol content was measured. Briefly, 500 mg samples from leaves which had been flash frozen in liquid nitrogen and stored at −80 °C, were ground in an ice-cold mortar with 50 mM sodium phosphate buffer (1:5; *w*:*v*; pH 7.0) containing: 1 mM EDTA, 0.5% PVP (polyvinylpyrrolidone), 0.5 M NaCl as well as SigmaFAST™ Protease Inhibitor Cocktail Tablet. The suspension was then centrifuged (20,000 ×*g*, 20 min) at 4 ° C. The supernatant was used for determination of POX, GST, CAT, TBARS, and protein content. Crude extracts were used for measuring α-tocopherol concentration.

#### 2.6.2. Enzymes Assay

Guaiacol peroxidase (POX EC 1.11.1.7) activity was assayed according to the method of Maehly [33]. Enzyme activity was estimated by measuring an increase in the absorbance of tetraguaiacol (ε = 26.6 mM/cm), a colored product of guaiacol oxidation, at 470 nm for 10 min at 30 °C. POX activity was expressed in units (1 U = 1 mmol of tetraguaiacol formed min/mg protein). Catalase (CAT, EC 1.11.1.6) activity was measured spectrophotometrically at 240 nm according to Dhindsa et al. [34] following decomposition of H_2_O_2_. The activity was calculated based on the extinction coefficient of ε = 45.2 M/cm. Enzyme activity was expressed in mmol H_2_O_2_ min/mg protein. Glutathione S-transferase (GST, EC 2.5.1.18) activity was determined with 1-chloro-2,4-dinitrobenzene (CDNB) as described by Habig et al. [35]. The activity was calculated from the increase in absorbance at 340 nm for one minute due to CDNB–GSH conjugation, with an extinction coefficient of 9.6 mM/cm. The enzyme activity was expressed in μmol 2,4-dinitrophenyl-S-glutathione min/mg protein.

#### 2.6.3. Thiobarbituric Acid Reactive Substance (TBARS) Content Assay

Lipid peroxidation was then measured in *P. filicifolia* shoots using the thiobarbituric acid (TBA) test, which determines MDA as an end product of lipid peroxidation [36]. Supernatants of *P. filicifolia* were incubated in water (at 98 °C) for one hour in a mixture 0.29 mM TBA and 10% acetic acid, and the resulting colored product (TBARS) was extracted with n-butanol. The organic layer was removed and its fluorescence was measured at 531 nm (excitation)/553 nm (emission). The concentration of lipid peroxides (nmol/mg protein) was calculated in terms of 1,1,3,3-tetraethoxypropane, which was used as a standard.

#### 2.6.4. Determination of α-Tocopherol

Tocopherol content was assayed according to Taylor et al. [37]. After saponification of the sample with 10 N KOH in the presence of 1.42 M ascorbic acid tocopherol was extracted to n-hexane. The fluorescence of the organic layer was measured at 280 nm (excitation) and 310 nm (emission). The concentration of α-tocopherol (μmol/mg protein) was calculated in terms of α-tocopherol, which was used as a standard.

#### 2.6.5. Native-Page Electrophoresis of Peroxidase

For POX Native-PAGE analysis, 0.5 g fresh weight plant material as described above, was homogenized at 4° C in a mortar, in 50-mM sodium phosphate buffer (1:5; *w*:*v*; pH 7.0) containing: 1 mM EDTA, 0.5% PVP, 0.5 M NaCl as well as SigmaFAST™ Protease Inhibitor Cocktail Tablet. The mixture was then centrifuged (20,000× *g*, 20 min; 4 °C). Native PAGE was then performed. Equal amounts of soluble proteins (20 mg) were loaded to each well. The protein concentration was determined according to Bradford [38]. POX activity as analysed by native PAGE at 4 °C,180 V, using 10 mM Tris-HCl buffer pH 8.3, containing 80 mM glycine without sodium dodecyl sulphate. POX bands were visualized on 8% polyacrylamide gels using the activity staining procedure. The gels were incubated in the staining buffer (50 mM acetate buffer pH 5.0, containing 10 mM pyrogallol) on the rocker-shaker, for 20 min in the dark at room temperature, and then hydrogen peroxide was added to a final concentration 0.1 %. The activities of POX isoform bands were calculated by determining band volume using Image Lab software (version 5.1, build 8, Bio-Rad Laboratories).

#### 2.6.6. Protein Determinations

Protein contents were measured as described by Bradford [38] using BSA as a standard.

### 2.7. Antioxidant Assays

The antioxidant capacity of *P. filicifolia* shoot extracts was determined by DPPH (2,2-diphenyl-1-picryhydrazyl) and ABTS•+ (2,2′-azino-di-(3-ethylbenzthiazoline sulfonic acid)) radical scavenging as described by Mareček et al. [39]. The 25 µL amounts of *P. filicifolia* shoot extracts diluted 10-fold in 80% methanol were mixed with 100 µL of DPPH or 50 µL of ABTS reagent. The samples and blanks were incubated in the darkness at room temperature for 30 min. and the absorbance was measured at 518 nm or 734 nm, respectively. A blank sample consisting of 25 µL 80% methanol instead of DPPH reagent, was included to correct for any sample absorbance at 518 nm. The mean absorbance for three replicates for each sample was calculated and expressed as mg of TROLOX equivalents (TE)/g DW using the regression equation determined from the standard curves: y = 0.0012x – 0.016, r^2^ = 0.9922 and y = 0,0011x − 0.0155 r^2^ = 0.9960, for ABTS and DPPH assays, respectively.

### 2.8. Statistical Analysis

All the analyses were performed with proper experimental and analytical replicates. The statistical significance between means was assessed using the Kruskal–Wallis one-way analysis of variance. All analyses were performed using STATISTICA 13.1 PL software. The probability of *p* < 0.05 was considered as significant. Pair-wise metabolite-antioxidant effects correlations were calculated by Pearson’s correlation coefficient test.

## 3. Results and Discussion

### 3.1. Effect of Culture Conditions on Polyscias filicifolia Shoot Growth

**Figure 1:**
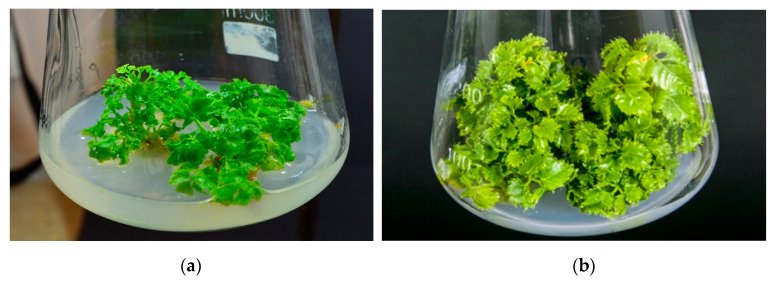

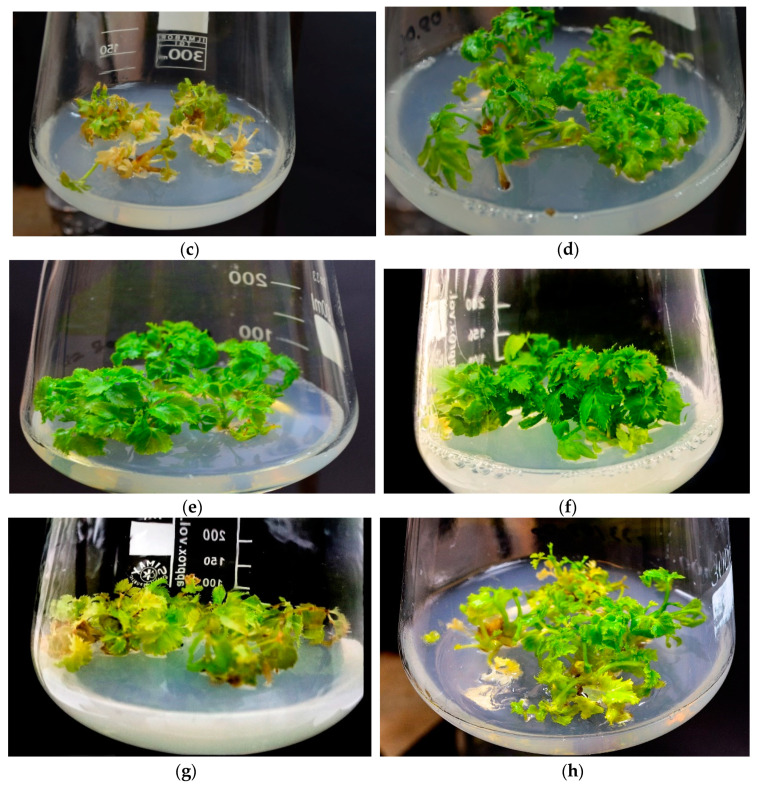
Morphology of *Polyscias filicifolia* shoots growing on LS medium supplemented with 8.88 µM BAP and 2.32 µM KIN for 4 weeks and then transferred to the fresh medium and cultivated for one week: (**a**) control cultures (untreated); cultures supplemented with elicitor/s and/or alarmone: (**b**) 50 µM SA; (**c**) 200 µM MeJA; (**d**) 50 µM SA + 200 µM MeJA; (**e**) 5 µM Ap3A; (**f**) 5 µM Ap3A + 50 µM SA; (**g**) 5 µM Ap3A + 200 µM MeJA; (**h**) 5 µM Ap3A + 50 µM SA + 200 µM MeJA.

The elicitors tested in the present work salicylic acid (SA), methyl jasmonate (MeJA) and alarmone compound Ap3A, used individually and in combinations, affected the rate of shoot multiplication, the amount of fresh and dry biomass and the length of the shoots (Table 1). The highest shoot multiplication rate (3.17 ± 0.08) was achieved for shoots of *P. filicifolia* grow on LS medium with the addition of Ap3A, however, this result was statistically significant only in comparison to the effect of MeJA, where the proliferation rate amounted to 2.05 ± 0.05 and was the lowest among all tested combinations. Similarly, MeJA treatment had a significant influence on shoot shortening in comparison to Ap3A. The other treatments were not found to significantly affect shoot proliferation, nor elongation (Table 1). In addition, the treatment type did not appear to have any statistically significant effect on fresh and dry biomass accumulation in relation to control cultures. This is the first study to examine the effect of Ap3A on plant growth parameters when applied exogenously; it is also the first report on the influence of Ap3A in combination with the elicitors MeJA and SA. Most of the applied treatments appeared to have a negligible effect on shoot growth parameters under the conditions of this study; however, MeJA has frequently been reported to have a detrimental effect on growth is due to mitosis inhibition and promotion of senescence (Figure 1) [2,40].

### 3.2. The Effect of Culture Conditions on the Production of Oleanolic Acid and Phenolic Compounds

The methanolic extracts of the cultured shoots were subjected to phytochemical analysis. The results indicate that all treatments resulted in significant increases in the accumulation of phenolic compounds and OA in relation to controls (Table 2; Figure S1). Elicitation with combined SA + MeJA resulted in a significantly (*p* < 0.05) higher yield of CGA, CA, FA, TPC and TFC compared to cultures where the elicitors were used individually. Among all the investigated phenolic acids (CGA, CA and FA) CGA was the most abundant with its highest content being found in shoots treated with SA + MeJA (4.18 mg/g DW), followed by Ap3A + SA + MeJA treatment (3.33 mg/g DW) or by SA alone (3.23 mg/g DW); however, these means were not statistically different (*p* < 0.05). Our present findings are consistent with those of Figat et al. [27], combined application of MeJA + SA stimulated phenolic compounds accumulation in *P. filicifolia* shoot cultures. However, in suspension cultures of *Psoralea coryfolia*, combined MeJA + SA treatment did not augment psoralen content in contradiction to their individual usage [41]. A similar effect was observed in suspension cultures of *Thevetia peruviana* [42]. A comparative 1H-NMR-based study of *T. peruviana* metabolome changes following MeJA, SA or MeJA + SA elicitation, found that MeJA most effectively augments phenolic compound production, followed by SA + MeJA joint treatment; in contrast, SA treatment alone did not significantly increase accumulation over control values [43]. Moreover, it was revealed that unlike the plants treated with SA alone or SA + MeJA, those treated with MeJA demonstrated a significant increase in glucose, glutamine and proline levels, and redirected carbon and energy flow toward de novo production of phenolic compounds. These findings contrast with those obtained in the present study, where combined SA + MeJA treatment elicited the greatest phenolic acid production and the highest total content of phenolic compounds and flavonoids (Table 2). This could be explained by the fact that different plant species were examined, and that shoot cultures were used in one study and cell suspension cultures in the other [3,44]. Similarly, Dong et al. [12] reported that SA supplementation improved phenolic compound production in cell suspension cultures of *Salvia miltiorrhiza*. Additionally, Abbasi et al. [45] described the stimulatory effect of SA on phenolic compounds biosynthesis in shoot cultures of *Ajuga integrifolia*. Similarly, SA appears to effectively stimulate andrographolide accumulation in shoot cultures of *Andrographis paniculata* [46]. Interestingly, in a study of *Hypericum perforatum*, SA treatment was also found to nearly double hypericin and pseudohypericin accumulation in cell suspension cultures, but not in shoot or callus culture [47].

In contrast, the present study, the simultaneous addition of Ap3A with SA or MeJA did not significantly increase the production of the investigated phenolic acids compared to the individual elicitors. However, in previous examinations Ap3A and cyclodextrins acted synergistically enhancing phenylpropanoid biosynthesis in *Vitis vinifera* cell suspension cultures [14]. In addition, in the present study, Ap3A treatment was found to have limited efficacy in enhancing OA biosynthesis was (Table 2). It is difficult to compare these findings with previous studies, as the production of OA has been investigated solely in cell suspension and hairy root cultures, even so, in these cases, jasmonic acid and MeJA appeared to be the most effective elicitors [48,49,50,51,52,53].

MeJA treatment has been found to significantly up-regulate the expression of genes engaged in OA biosynthesis in embryogenic callus cultures of *Gentiana straminea*, while SA was ineffective [54]. Next, the analysis of transcription response to MeJA application to *Ocimum basilicum* plants revealed up-regulation of two oxidosqualene cyclase genes: *ObAS1* (responsible for the production of β-amyrin) and *ObAS2* (responsible for the production of both α-amyrin and β-amyrin). The expression of these genes was the highest in inflorescences and leaves (higher in older than younger ones), and the lowest in roots [55]. Both α-amyrin and β-amyrin are direct precursors of oleane- and ursane-type triterpenes. In addition, both MeJA and yeast extract was found to enhance OA biosynthesis in *Achyranthes bidentata* cell suspension cultures and up-regulated the expression of 3-hydroxy-3-methylglutaryl coenzyme A reductase (HMGR) which is a key enzyme of the mevalonate pathway for isoprenoid biosynthesis [56,57], especially under stress conditions [58].

In the current study, no negative cross-talk was found between SA and MeJA regarding the induction of biosynthetic pathways. Despite this, they nevertheless demonstrated a synergetic effect. This is probably due to the two elicitors being applied at concentrations that allowed a favourable response to detrimental environmental factors by enhancing secondary metabolites production [59].

### 3.3. The Effect of Culture Conditions on the Antioxidant Activity of P. filicifolia Shoot Extracts

The antioxidant properties of methanol extracts obtained from the treated *P. filicifolia* shoots were assessed by DPPH and ABTS radical scavenging methods. The results were converted to Trolox values [mg TE/g DW], a water-soluble tocopherol analogue used as the antioxidant standard. It was found that elicitation significantly (*p* < 0.05) increased the antioxidant activity of the studied extracts (Table 2). The highest antioxidant activity determined by DPPH assay, was demonstrated for the extracts of *P. filicifolia* shoots cultivated with SA + MeJA, and this value amounted to 30.85 mg TE/g DW and was 3.7-fold higher than control values. Only slightly lower DPPH scavenging abilities were demonstrated by the shoots treated with SA (28.68 mg TE/g DW). Interestingly, for the ABTS assay, the shoots elicited with SA alone demonstrated the greatest scavenging potential (16.63 mg TE/g DW), followed by those treated with Ap3A + SA (16.30 mg TE/g DW). The reported radical scavenging potentials were closely correlated with the content of investigated phenolic acids. Pearson’s correlation coefficient amounted to r^2^ = 0.81, 0.64, 0.80, 0.77, 0.87 and 0.64, for CGA, FA, CA, TPC, TFC and OA concentration, respectively. These results are consistent with those reported by Figat et al. [27] who note that the extract from shoots treated with SA + MeJA exhibited the strongest antioxidant activity, which could be attributed to the presence of phenolic compounds with strong antiradical properties [60]. Moreover, UHPLC-DAD-MS/MS analysis of SA + MeJA extracts revealed presence of ferulic acid and its derivatives, which are known to be effective scavengers of [61]. The strong antioxidant properties of the extracts prepared from shoots subjected to SA + MeJA elicitation are consistent with their chemical profile which revealed high concentrations of the investigated phenolic acids, TPC and TFC and triterpenoid compound—OA. These compounds have been found to possess significant antioxidant activity [23].

### 3.4. The Effect of Selected Elicitors and Their Combinations on Protein Concentration and Lipid Peroxidation

**Figure 2:**
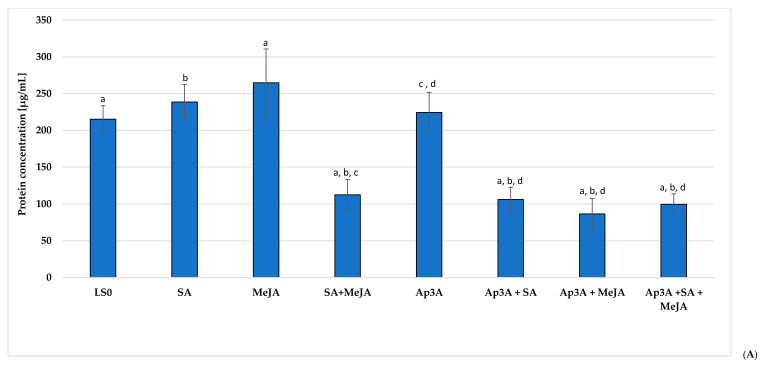

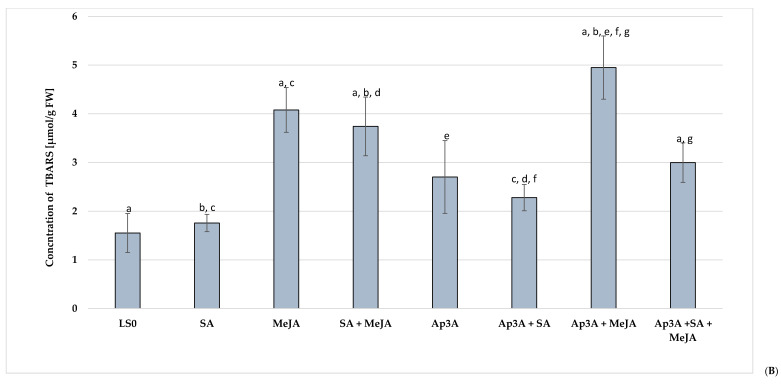

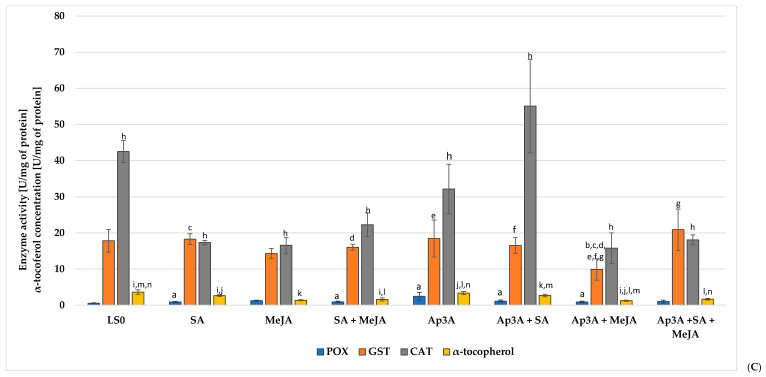
The influence of different modifications of LS medium supplemented with 8.88 µM BAP and 2.32 µM KIN on *Polyscias filicifolia* shoots: (**A**) protein level in extracts; (**B**) levels of lipid peroxidation; (**C**) activity of antioxidant enzymes and levels of non-enzymatic antioxidant α-tocopherol. Means denoted with the same letter were statistically significant (*p* < 0.05).

The highest protein concentration was achieved for shoots grown on medium with the addition of MeJA (287.80 µg/mL) and was higher by 13.38% compared to control shoots (Figure 2A). Although, slightly lower protein concentrations were obtained in the presence of SA (238.70 µg/mL), these differences were not statistically significant (Figure 2A). Much lower protein levels were observed for combined elicitation. Elicitation with SA + MeJA, Ap3A + SA, Ap3A + MeJA and Ap3A + SA + MeJA, yielded protein concentrations ranging from 99.70 to 112.50 µg/Ml. These values are almost 50% lower than control shoots (Figure 2A). In contrast, Bhattacharyya et al. report similar protein levels between *Podophyllum hexandrum* suspension cultures elicited with MeJA and untreated controls; however, the protein’s profile varied. The authors reported the higher levels of proteins involved in the phenylpropanoid biosynthetic pathway and the stress response [62].

Malondialdehyde (MDA) is commonly used as an indicator of oxidative damage to lipid membranes, with a high level reflecting an oxidative imbalance. When reacted with 2-thiobarbituric acid, TBARS a colorful, adduct that can be measured with spectrophotometry. In the present study, the level of oxidative damage was calculated by two methods and finally presented as μmol TBARS /g of FW of *P. filicifolia* shoots (Figure 2B). Except for all elicitor treatments were found to increase TBARS content. Although all Ap3A treatments appeared to increase lipid peroxidation, the highest TBARS concentration was observed following Ap3A + MeJA treatment, i.e. with levels over four-fold higher than control values; this may indicate a high degree of damage to lipid membranes components and high levels of ROS production. The high levels of TBARS observed in our experiments may indicate that the balance was shifted towards pro-oxidative reactions possibly due to an ineffective antioxidative system and/or high activity of lipoxygenases. ROS, are produced in the early stages of plant responses to various stress factors, and these play a crucial role in the signaling network [9]. One of the stress-induced ROS, H_2_O_2_, also plays an important regulatory role in lignin biosynthesis. Pietrowska-Borek et al. [13,14] report that Ap3A, applied alone or in combination with cyclodextrins increased the transcript level of genes encoding key phenylpropanoid-pathway enzymes in *Vitis vinifera* suspension cells cultures; in addition, treatment also increased the production of trans-resveratrol inside cells and its secretion into the extracellular medium. Furthermore, exogenous application of Ap3A to *A. thaliana* seedlings markedly induced the expression of genes encoding phenylalanine ammonia lyase and 4-coumarate CoA ligase enzymes, which play a role in the phenylpropanoid pathway. It is believed that plants are able to regulate ROS concentrations according to their current need [8], by developing efficient ROS detoxifying systems based on enzymatic antioxidants like POX and CAT, and non-enzymatic ones like glutathione, ascorbic acid and tocopherols [10,63,64].

In the present study, significant differences in CAT and POX activities were observed between *P. filicifolia* shoots, growing under control conditions and those treated with alarmone and elicitors (Figure 2C). Generally, CAT activity significantly decreased in response to used elicitors, however it was 24% higher than control values in the presence of Ap3A + SA. The lowest CAT activities, i.e. approximately 60% lower than control values were noticed in shoots elicited individually with SA alone, MeJA alone and MeJA in combination with another elicitor. The decrease observed in CAT activity following SA treatment may be connected with its inhibitory effect on CAT in plant cells.

The reverse tendency was observed for POX, with increased activity being found in all elicited variants, i.e. approximately. 40 to 130% than control values. The highest POX activity was in shoots treated with Ap3A, with (values around 137% higher than controls. Additionally, Native-PAGE identified the presence of extra POX isoforms with pyrogallol used as an electron donor in the variants treated with SA, Ap3A and Ap3A + SA (Figure S2).

Peroxidases are oxidoreductases class enzymes, which participate in both H_2_O_2_ scavenging H_2_O_2_ generation. H_2_O_2_ is believed to play a direct role in the reaction of plants to pathogen attack, and the rearrangement of cell wall polymers, and may also participate in signaling networks [10]. The presence of H_2_O_2_ in the plant response to stress conditions implicates also the induction of GST, which is involved in the detoxification of lipid peroxides formed during oxidative stress [8]; however, in the current study there was no correlation in GST activity and lipid oxidation levels (Figure 2B,C), probably due to the short time of elicitation, which was not sufficient to activate the expression of relevant enzyme genes. However, high GST activity was detected the SA, Ap3A and Ap3A+SA+MeJA cultures (Figure 2C) which implied the participation of ROS in cell signaling.

The highest levels of α-tocopherol, a non-enzymatic antioxidant engaged in quenching singlet oxygen [11], were found in the control cultures and those treated with Ap3A, followed by those treated with SA and SA+Ap3A (Figure 2C). This observation coincided with a high antioxidant enzyme activity. It is possible that in these circumstances, α-tocopherol could play a role in ameliorating the effects of stress exerted by the transfer of shoots to the fresh medium, which would entail a greater demand for antioxidative compounds [65]. The lowest α-tocopherol level was detected in plants elicited with MeJA alone or in combination with other compounds, with these findings being about 50–60% lower than in control. In plants, α-tocopherol plays an important role in the antioxidant system; indeed. Munné-Bosch et al. [64] propose that tocopherol is not only involved in the regulation of the cell redox homeostasis but can influence cell signaling by modulating jasmonate levels. As suggested by Schaller [66] α-tocopherol probably regulates the amount of MeJA in leaves by controlling lipid peroxidation in these organs.

It was observed that Ap3A considerably enhanced the production of ROS, to an even higher extent than the most efficient, in the current study, elicitors SA + MeJA; however, this did not translate into an elevated yield of secondary metabolites. This effect could be a result from negative feedback regulation, where jasmonate down-regulate its own concentration in jasmonate-regulating signaling pathway [67].

Our present findings illustrate the complexity of the varied signaling pathways activated in plant cells in response to environmental stimuli. However, further investigation is necessary to elucidate the role of alarmone in secondary metabolite production.

## 4. Conclusions

Our findings indicate that combined elicitation of *Polyscias filicifolia* shoots with 50 µM SA + 200 µM MeJA proved to be the most efficient and sufficient approach for enhanced accumulation of phenolic compounds and OA. Alarmone Ap3A did not enhance the biosynthesis of investigated compounds when used alone and exerted only a moderate effect when used together with other elicitors. Ap3A considerably enhanced the production of ROS, suggesting that ROS are engaged in signal transduction following Ap3A application. However, its high ROS production in turn did not translate into higher secondary metabolites production, probably due to the negative feedback regulation existing in plant cells.

## Figures and Tables

**Table 1:** The effect of culture conditions on the growth of *Polyscias filicifolia* shoots of cultivated in vitro under various culture conditions.

Type of Culture	Shoot Fresh Biomass Increase	Shoot Dry Biomass Increase	Shoot Multiplication Rate	Length of Shoots [cm]
LS0	2.37 ± 0.09	0.27 ± 0.05	2.02 ± 0.12	4.07 ± 0.16
LS_SA_	2.56 ± 0.11	0.37 ± 0.05	2.11 ± 0.07	4.30 ± 0.18
LS_MeJA_	1.72 ± 0.13	0.27 ± 0.04	2.05 ± 0.05 ^a^	3.40 ± 0.05 ^a^
LS_SA + MeJA_	3.10 ± 0.11	0.33 ± 0.04	2.25 ± 0.11	4.18 ± 0.03
LS_Ap3A_	3.06 ± 0.28	0.43 ± 0.03	3.17 ± 0.08 ^a^	4.62 ± 0.03 ^a^
LS_Ap3A + SA_	2.39 ± 0.15	0.25 ± 0.04	2.67 ± 0.08	4.51 ± 0.03
LS_Ap3A + MeJA_	2.02 ± 0.17	0.30 ± 0.05	2.60 ± 0.23	4.32 ± 0.04
LS_Ap3A +SA + MeJA_	3.10 ± 0.12	0.23 ± 0.07	2.27 ± 0.12	4.04 ± 0.04

LS0—control shoots were cultured without elicitors on LS medium supplemented with 8.88 µM BAP and 2.32 µM KIN. LS_SA_—LS medium supplemented with salicylic acid (SA) 50 µM. LS_MeJA_ —LS medium supplemented with methyl jasmonate (MeJA) 200 µM. LS_SA+MeJA_—LS medium supplemented with SA (50 µM) + MeJA (200 µM). LS_Ap3A_—LS medium supplemented with 5 μM Ap_3_A. LS_Ap3A+SA_—LS medium supplemented with two elicitors: Ap_3_A (5 μM) + SA (50 μM). LS_Ap3A+MeJA_—LS medium supplemented with two elicitors: Ap_3_A (5 μM) + MeJA (200 μM). LS_Ap3A+SA+MeJA_—LS medium supplemented with three elicitors: Ap_3_A (5 μM) + SA (50µM) + MeJA (200 μM). Data represent mean values ± SD from three independent experiments. Data within groups denoted with the same letters are statistically significant (*p* ˂ 0.05).

**Table 2:** The content of phenolic compounds and oleanolic acid in methanolic extracts from shoots *Polyscias filicifolia* growing under various culture conditions and their antioxidant potential.

Culture Modification	CGA [mg/g DW]	CA [mg/g DW]	FA [mg/g DW]	OA [mg/g DW]	Total Phenolics Content (TPC) [mg GAE/g DW]	Total Flavonoid Content (TFC) [mg QE/g DW]	ABTS [mg TE/g DW]	DPPH [mg TE/g DW]
LS0	1.09 ± 0.04	0.58 ± 0.13	0.02 ± 0.04 ^a^	0.11 ± 0.31	1.63 ± 0.06	1.21 ± 0.33	10.78 ± 0.09	8.28 ± 0.32
LS_SA_	3.23 ± 0.02 ^a^	0.79 ± 0.22 ^a^	0.07 ± 0.08 ^b^	0.25 ± 0.08	4.13 ± 0.22 ^a^	2.94 ± 0.02	16.63 ± 0.11	28.68 ± 0.49 ^a^
LS_MeJA_	2.31 ± 0.32 ^b^	0.77 ± 0.29 ^b^	0.09 ± 0.01 ^b^	0.17 ± 0.42	5.22 ± 0.05	3.79 ± 0.03 ^a^	15.36 ± 0.11 ^a^	26.88 ± 0.47 ^b^
LS_SA + MeJA_	4.18 ± 0.16	0.89 ± 0.23 ^c^	0.11 ± 0.11 ^c,e^	0.39 ± 0.41	7.62 ± 0.09	4.90 ± 0.02	14.46 ± 0.01	30.85 ± 0.52
LS_Ap3A_	2.18 ± 0.12 ^b^	0.92 ± 0.01 ^c^	0.04 ± 0.15 ^a,d^	0.18 ± 0.03	3.50 ± 0.12	4.13 ± 0.15 ^a,b^	14.86 ± 0.01 ^b^	24.64 ± 1.06 ^a,b^
LS_Ap3A + SA_	2.68 ± 0.18	0.86 ± 0.01	0.04 ± 0.18 ^a,d^	0.27 ± 0.15	4.11 ± 0.11 ^a^	3.65 ± 0.10 ^a^	16.30 ± 0.02	24.41 ± 0.79 ^c^
LS_Ap3A + MeJA_	2.38 ± 0.16 ^b^	0.91 ± 0.17 ^c^	0.09 ± 0.08 ^b,e,f^	0.19 ± 0.23	7.78 ± 0.09	3.57 ± 0.03 ^a^	15.38 ± 0.07 ^a^	24.76 ± 0.32 ^c^
LS_Ap3A +SA + MeJA_	3.33 ± 0.30 ^a^	0.79 ± 0.16 ^a,b,c^	0.10 ± 0.02 ^b,c,f^	0.34 ± 0.56	5.75 ± 0.02	4.46 ± 0.07 ^b^	14.88 ± 0.03 ^b^	26.98 ± 0.11 ^b^

LS0—control shoots were cultured without elicitors on LS medium supplemented with 8.88 µM BAP and 2.32 µM KIN. LS_SA_—LS medium supplemented with salicylic acid (SA; 50 µM). LS_MeJA_—LS medium supplemented with methyl jasmonate (MeJA; 200 µM). LS_SA+MeJA_—LS medium supplemented with SA (50 µM) + MeJA (200 µM). LS_Ap3A_—LS medium supplemented with diadenosine 5′,5‴-*P*^1^*P*^3^-triphosphate (Ap3A; 5 μM). LS_Ap3A+SA_—LS medium supplemented with Ap3A (5 μM) + SA (50 μM). LS_Ap3A+MeJA_—LS medium supplemented with Ap3A (5 μM) + MeJA (200 μM). LSAp3A+SA+MeJA—LS medium supplemented with three elicitors: Ap3A (5 μM) + SA (50µM) + MeJA (200 μM). Data are expressed as means ± SD from three independent experiments. Means within the group denoted with the same letter were not statistically significant (*p* ˂ 0.05). The differences between other means were statistically significant (*p* ˂ 0.05).

## Data Availability

The data presented in this study are available on request from the corresponding author. The data are not publicly available because they are a part of one of co-authors habilitation work and after finishing this work the data will be accessible.

## Supplementary Materials

The following are available online at https://www.mdpi.com/2073-4409/10/2/419/s1, Figure S1: sample HPLC chromatograms. Figure S2: Native-PAGE POX gel demonstrating additional isoforms of peroxidase (where pyrogallol was used as an electron donor) in SA, Ap3A and Ap3A+SA variants.
