# Supplementary material for: In Vitro Response of Polyscias filicifolia (Araliaceae) Shoots to Elicitation with Alarmone–Diadenosine Triphosphate, Methyl Jasmonate, and Salicylic Acid
Source: Cells. 2021 Feb 17;10(2):419. doi: 10.3390/cells10020419 (PMC7922777; doi:10.3390/cells10020419)
Supplement: Supplementary file 1 [file cells-10-00419-s001.pdf]

*Article*

# In Vitro Response of *Polyscias filicifolia* (Araliaceae) Shoots to Elicitation with Alarmone–Diadenosine Triphosphate, Methyl Jasmonate, and Salicylic Acid

**Anita Śliwińska [1], Marcin R. Naliwajski [2,*], Agnieszka Pietrosiuk [1] and Katarzyna Sykłowska-Baranek [1]**

[1] Department of Pharmaceutical Biology and Medicinal Plant Biotechnology, Faculty of Pharmacy, Medical University of Warsaw, 1 Banacha, 02-097 Warsaw, Poland; anita.sliwinska@wum.edu.pl (A.S.); agnieszka.pietrosiuk@wum.edu.pl (A.P.), katarzyna.syklowska-baranek@wum.edu.pl (K.S.-B.)

[2] Department of Plant Physiology and Biochemistry, Faculty of Biology and Environmental Protection, University of Lodz, ul. Banacha 12/16, 90-237 Lodz, Poland

* Correspondence: marcin.naliwajski@biol.uni.lodz.pl

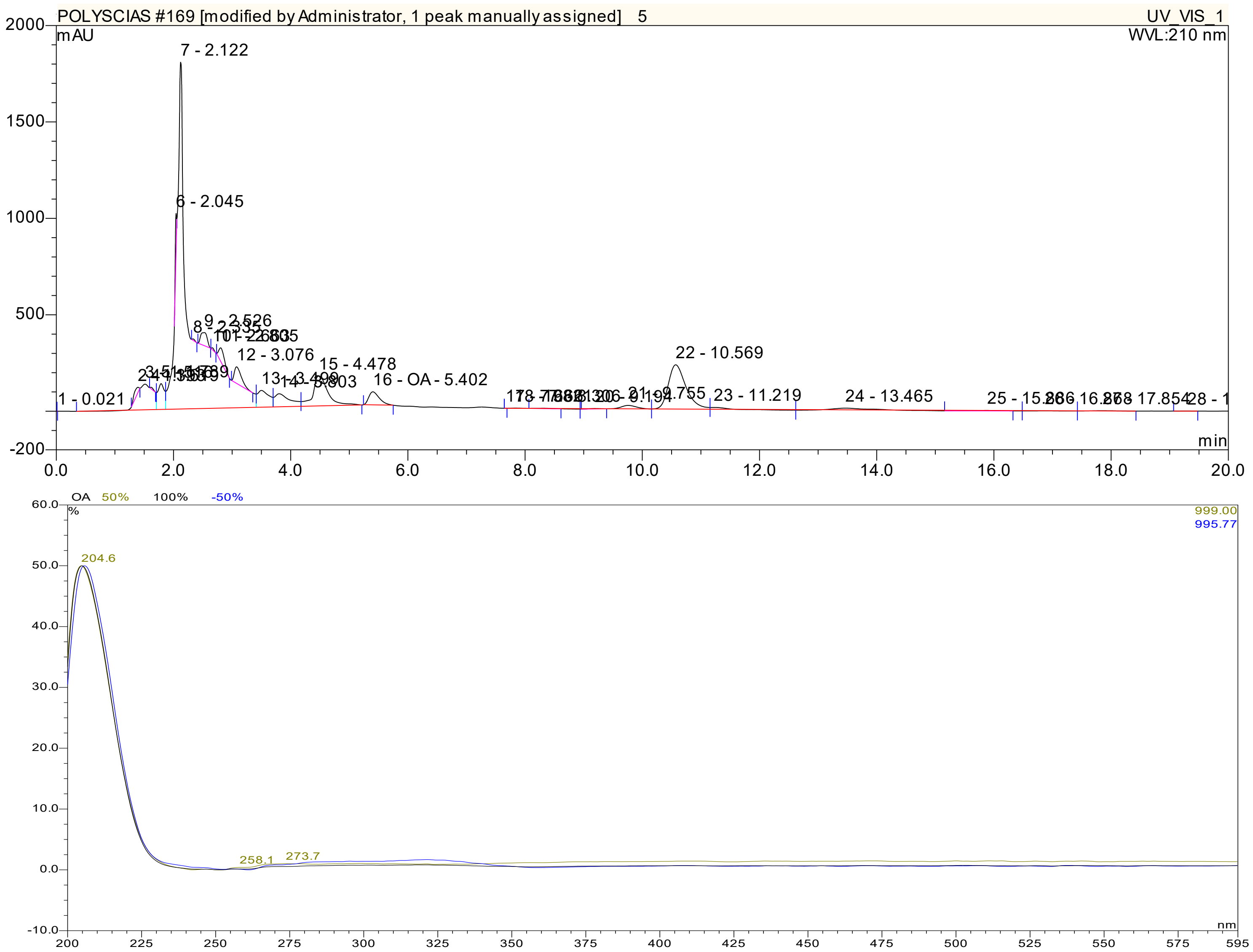
POLYSCIAS #169 [modified by Administrator, 1 peak manually assigned] 5
UV_VIS_1
WVL:210 nm
mAU
2000
1500
1000
500
-200
7 - 2.122
6 - 2.045
12 - 3.076
15 - 4.478
16 - OA - 5.402
22 - 10.569
23 - 11.219
24 - 13.465
1 - 0.021
min
0.0
2.0
4.0
6.0
8.0
10.0
12.0
14.0
16.0
18.0
20.0
OA 50% 100% -50%
%
999.00
995.77
204.6
258.1
273.7
60.0
50.0
40.0
30.0
20.0
10.0
0.0
-10.0
nm
200
225
250
275
300
325
350
375
400
425
450
475
500
525
550
575
595
(a)
(b)

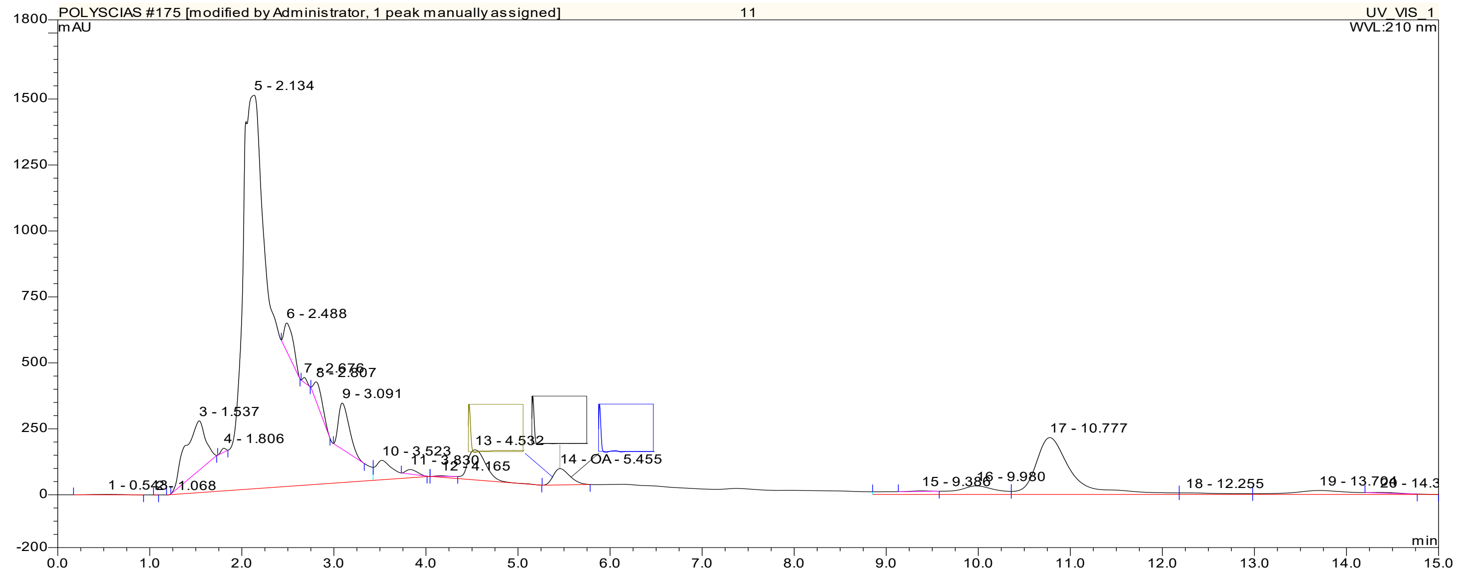

POLYSCIAS #175 [modified by Administrator, 1 peak manually assigned]
11
UV_VIS_1
WVL:210 nm
mAU
5 - 2.134
6 - 2.488
3 - 1.537
4 - 1.806
9 - 3.091
10 - 3.523
13 - 4.532
14 - OA - 5.455
17 - 10.777
18 - 12.255
min


(c)

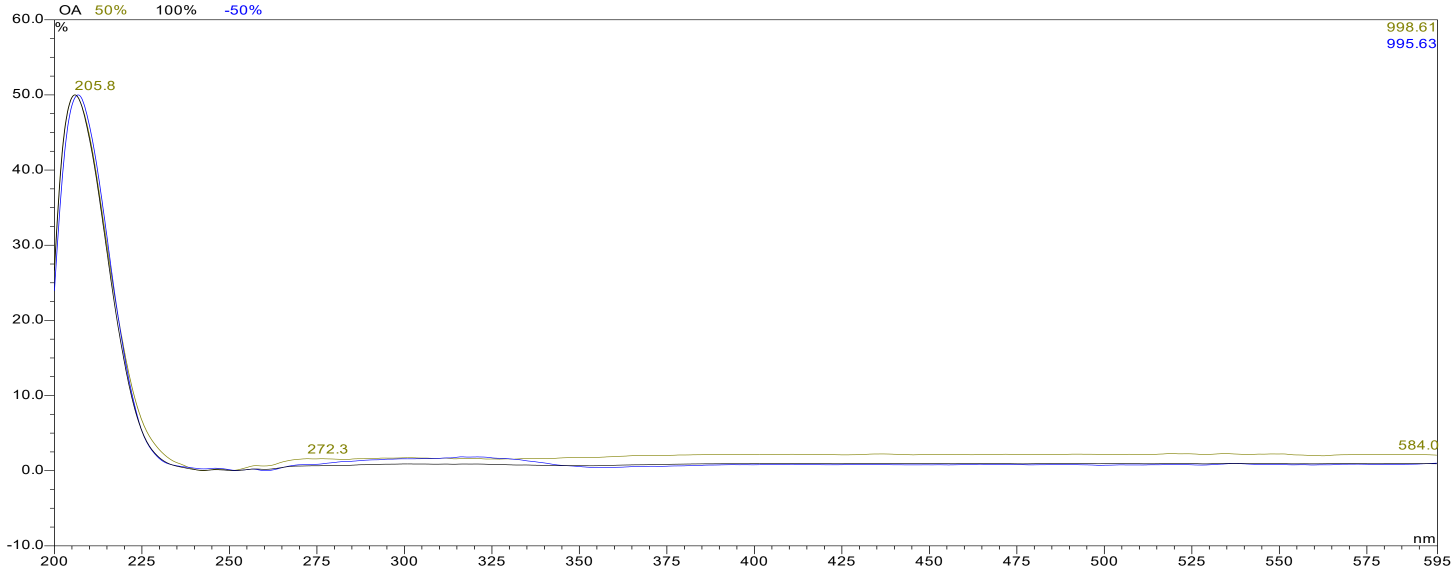

OA 50% 100% -50%
%
998.61
995.63
205.8
272.3
584.0
nm


(d)

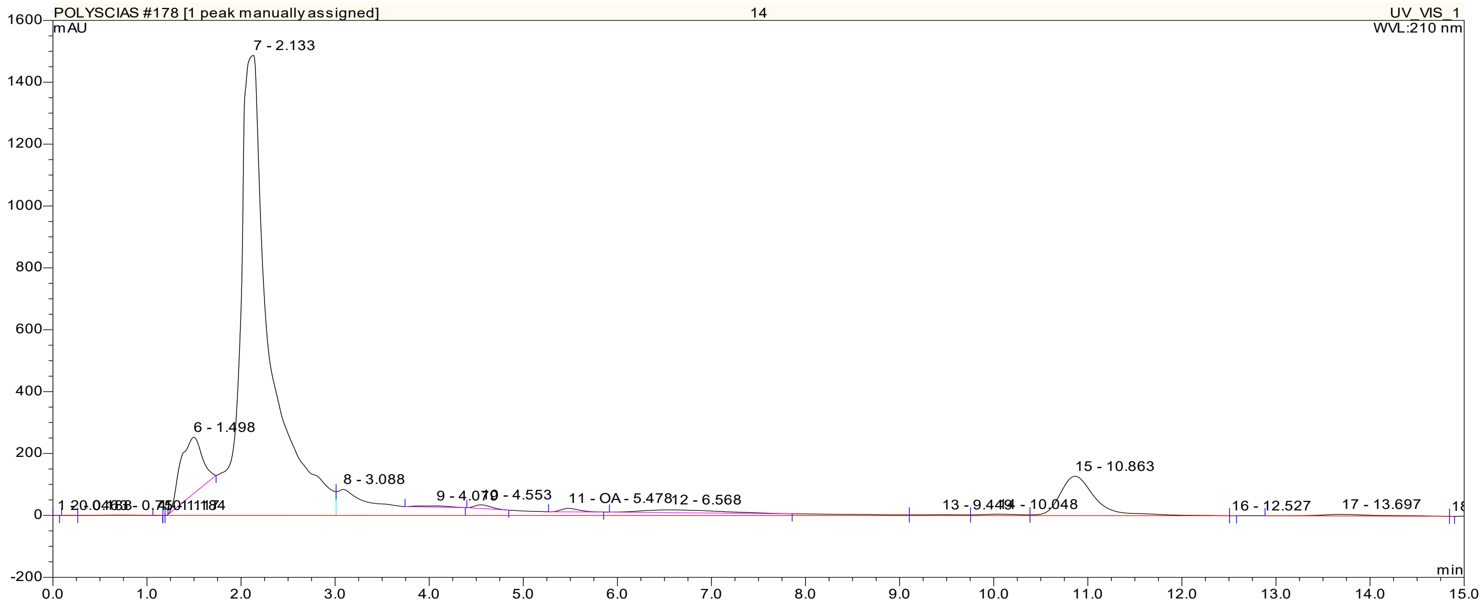
POLYSCIAS #178 [1 peak manually assigned]
14
UV_VIS_1
WVL:210 nm
mAU
7 - 2.133
6 - 1.498
8 - 3.088
10 - 4.553
11 - OA - 5.478
12 - 6.568
14 - 10.048
15 - 10.863
16 - 12.527
17 - 13.697
min

(e)

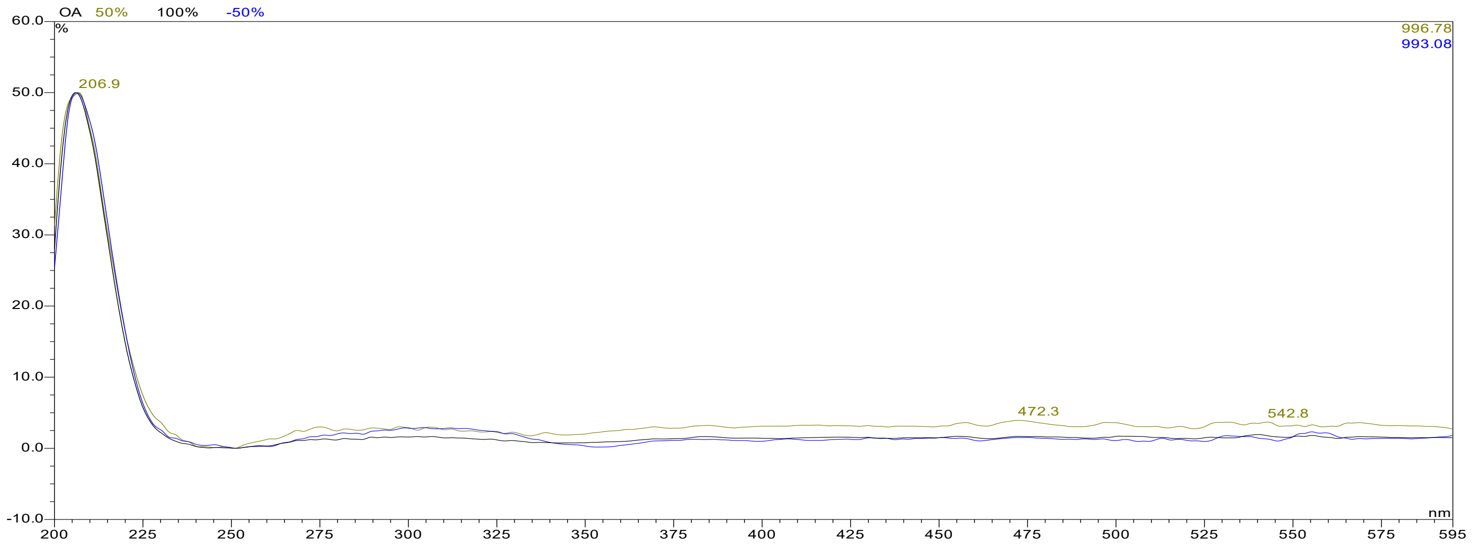
OA 50% 100% -50%
%
206.9
996.78
993.08
472.3
542.8
nm

(f)

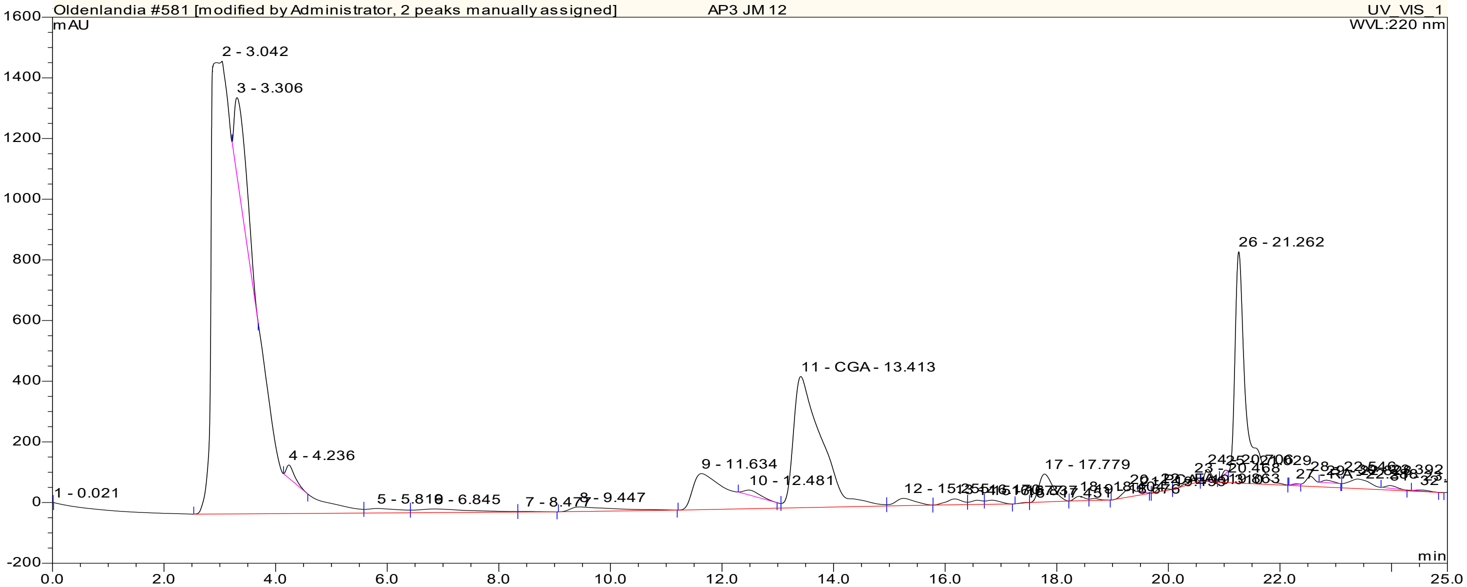
Oldenlandia #581 [modified by Administrator, 2 peaks manually assigned]
AP3 JM 12
UV_VIS_1
WVL:220 nm
mAU
min
1 - 0.021
2 - 3.042
3 - 3.306
4 - 4.236
11 - CGA - 13.413
9 - 11.634
10 - 12.481
17 - 17.779
26 - 21.262

(g)

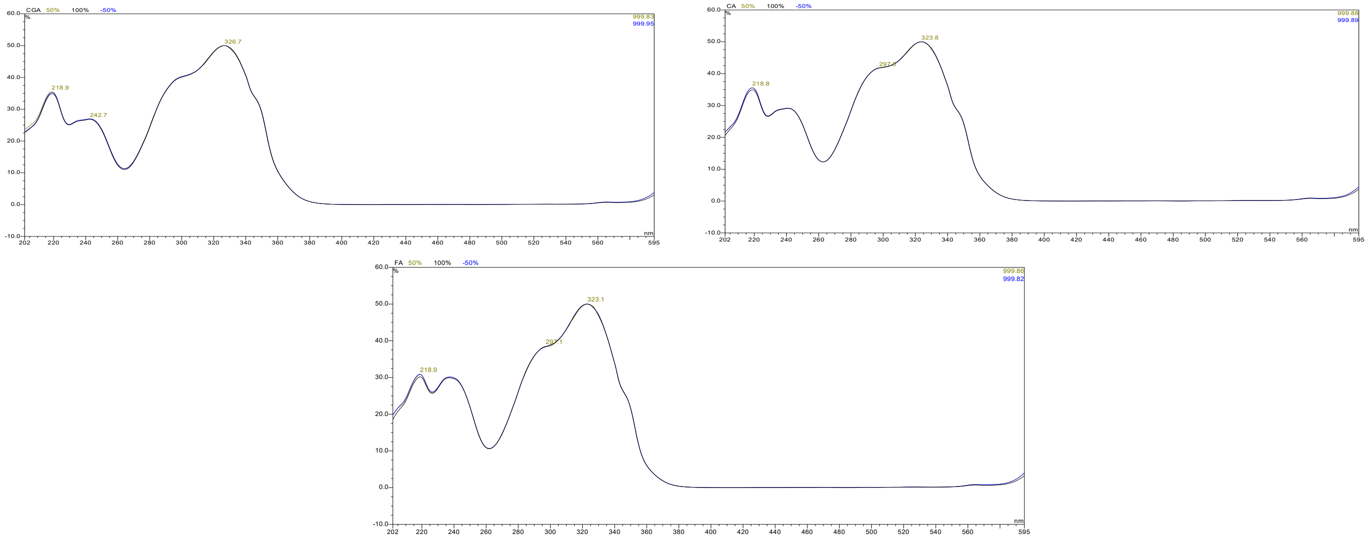
CGA
CA
FA
nm

(h)

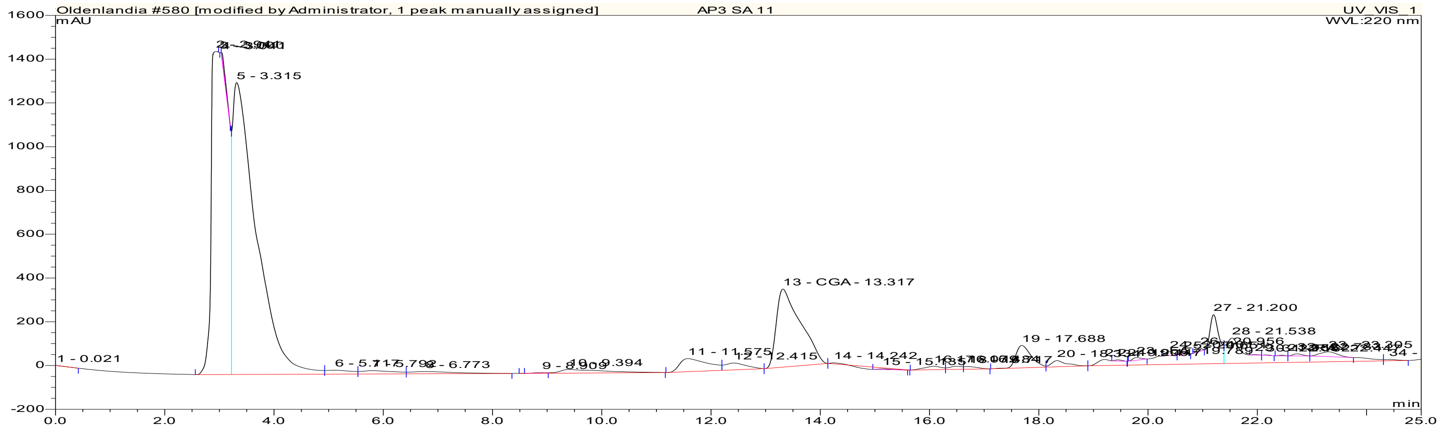


(i)

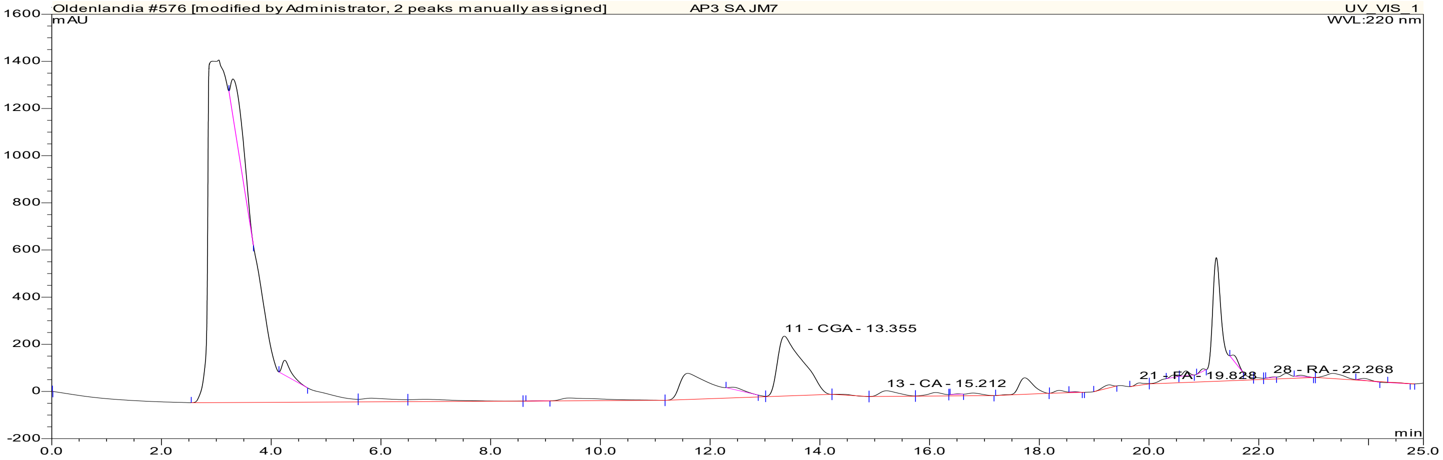


(j)

**Figure 1S.** Sample HPLC chromatograms resulted from HPLC-DAD-UV-Vis analysis; (**a**) hydrolysed methanolic extract from shoots cultivated in presence of 50 µM SA and (**b**) oleanolic acid's spectrum (λ=210 nm); (**c**) hydrolysed methanolic extract from shoots cultivated in presence of 5 µM Ap3A + 50 µM SA and (**d**) oleanolic acid's spectrum; (**e**) hydrolysed methanolic extract from shoots cultivated in presence of 5 µM Ap3A + 200 µM MeJA and (**f**) oleanolic acid's spectrum; (**g**) methanolic extract from shoots cultivated in presence of 5 µM Ap3A + 200 µM MeJA and (**h**) chlorogenic, caffeic and ferulic acids' spectra (λ=220 nm); (**i**) methanolic extract from shoots cultivated in presence of 5 µM Ap3A + 200 µM MeJA; (**j**) methanolic extract from shoots cultivated in presence of 5 µM Ap3A + 50 µM SA + 200 µM MeJA.

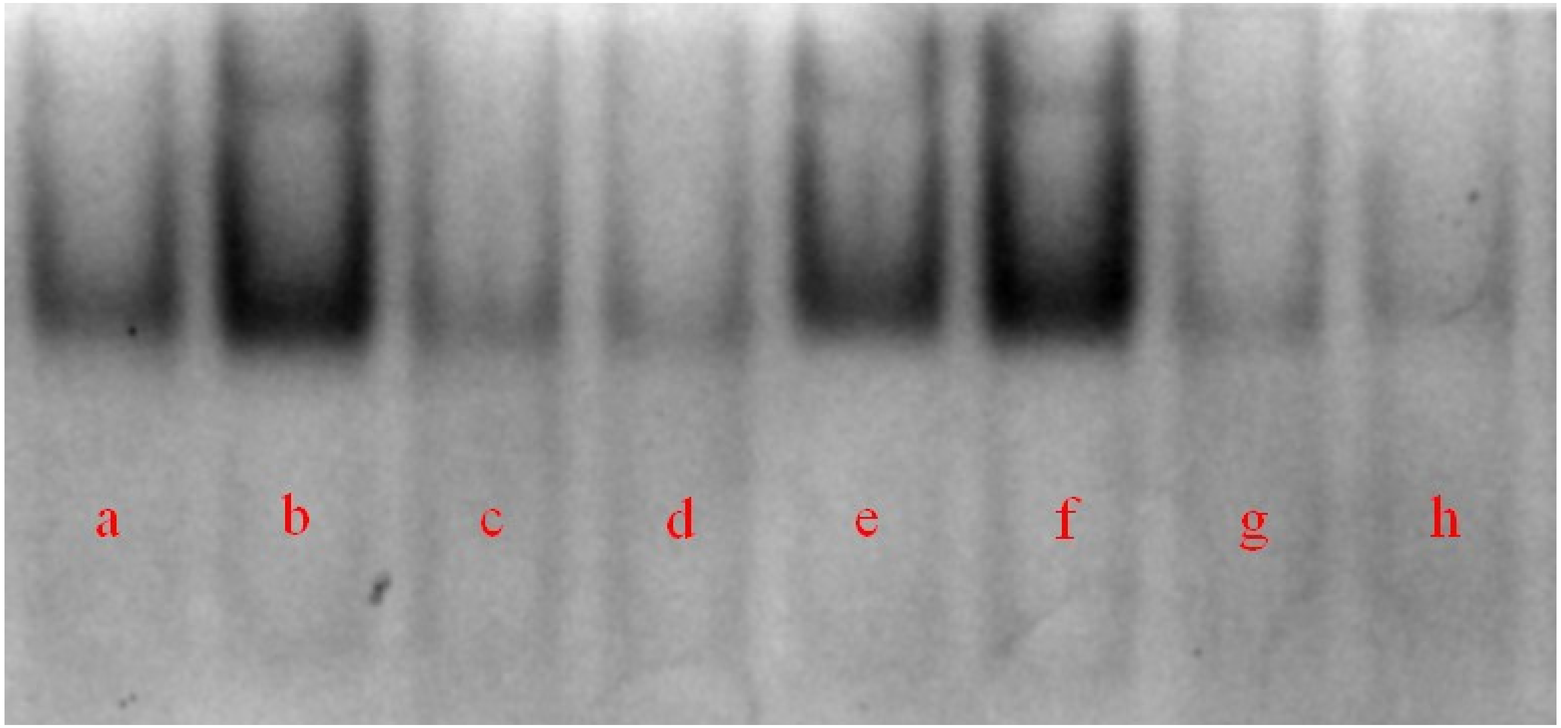

**Figure S2.** Native-PAGE POX gel demonstrating additional isoforms of peroxidase (where pyrogallol was used as an electron donor) in SA, Ap3A and Ap3A+SA variants: (**a**) control cultures (untreated); and cultures supplemented with elicitor/s and/or alarmone: (**b**) 50 µM SA; (**c**) 200 µM MeJA; (**d**) 50 µM SA + 200 µM MeJA; (**e**) 5 µM Ap3A; (**f**) 5 µM Ap3A + 50 µM SA; (**g**) 5 µM Ap3A + 200 µM MeJA; (**h**) 5 µM Ap3A + 50 µM SA + 200 µM MeJA.
